# ^18^F-FDG PET/CT assists the diagnosis of primary pancreatic lymphoma: Two case reports and literature review

**DOI:** 10.3389/fmed.2024.1370762

**Published:** 2024-02-23

**Authors:** Jian Wang, Yujing Zhou, Hongwei Liu, Jianli Zhou, Xin Li

**Affiliations:** ^1^Department of Nuclear Medicine, Qilu Hospital of Shandong University, Jinan, China; ^2^Department of Nuclear Medicine, Dezhou People’s Hospital, Dezhou, China

**Keywords:** primary pancreatic lymphoma, ^18^F-fluorodeoxyglucose positron emission tomography/computed tomography, case report, pancreatic carcinoma, therapeutic efficacy

## Abstract

Primary pancreatic lymphoma (PPL) is a rare malignancy, which is defined as a mass centered in pancreas with involvement of contiguous lymph nodes and distant spread may exist. Accurate diagnosis of PPL prior to pathological confirmation remains challenging, underscoring the critical significance of preoperative imaging assessments. This case report collected two instances of PPL that underwent initial evaluation via ^18^F-fluorodeoxyglucose positron emission tomography/computed tomography (^18^F-FDG PET/CT) between August 2021 and July 2022. Correspondingly, pertinent literature encompassing ^18^F-FDG PET/CT data related to PPL was meticulously reviewed. Including our aforementioned pair of cases, a cumulative total of 25 instances of PPL were assembled. The distinctive profile of ^18^F-FDG PET/CT images of PPL predominantly manifests as hypermetabolic lesions with diminished density. Primarily characterized by singular lesions and comparatively substantial volumetric dimensions, a total of eleven cases revealed contiguous lymph node engagement, with five instances displaying distant dissemination encompassing lymph nodes in multiple locations. Amongst these, ten patients underwent sequential ^18^F-FDG PET/CT follow-up post-intervention. In comparison to pancreatic carcinoma, PPL lesions exhibited heightened hypermetabolism, augmented volumetric proportions, and distinct patterns of distant metastasis. This study indicates that the pivotal role of ^18^F-FDG PET/CT in the diagnosis and assessment of therapeutic efficacy in PPL is unequivocal. Combined with the clinical attributes of patients, the integration of ^18^F-FDG PET/CT augments the differential diagnostic capacity differentiating PPL from pancreatic carcinoma.

## 1 Introduction

Primary pancreatic lymphoma (PPL) constitutes a rare malignancy, with an incidence rate of less than 2% among extranodal non-Hodgkin lymphomas in populations with good immunocompetent (1). The World Health Organization (WHO) has defined PPL as predominantly lymphomatous proliferation centered within the confines of the pancreas. However, involvement of contiguous lymph nodes within the pancreatic region and the possibility of distant dissemination remain plausible (2, 3), with potential associations to immunodeficiency as an underlying etiological factor. Owing to its rarity and a lack of distinct clinical manifestations, PPL poses a considerable diagnostic challenge prior to histopathological assessment, frequently leading to misdiagnoses akin to other pancreatic space-occupying lesions, such as pancreatic carcinoma (4).

Functional imaging stands as a pivotal achievement in the realm of medical imaging, enabling the simultaneous provision of metabolic and anatomical insights. Foremost among these modalities, ^18^F-fluorodeoxyglucose positron emission tomography/computed tomography (^18^F-FDG PET/CT) has emerged as a widely embraced functional imaging technique. It assumes a pivotal role in discerning differential diagnoses, facilitating staging procedures, and monitoring the trajectory of lymphomas (5, 6). However, owing to the infrequent occurrence of PPL, there exists a dearth of literature detailing the distinctive ^18^F-FDG PET/CT features pertinent to PPL. Notably, no comprehensive systematic review has hitherto encapsulated the value of ^18^F-FDG PET/CT in the context of PPL.

We report two PPL cases that underwent assessment via 18F-FDG PET/CT and discuss the potential utility of ^18^F-FDG PET/CT in the realm of PPL through a literature review. It is pertinent to underscore that this retrospective study has been carried out in accordance with the Declaration of Helsinki (2000) of the World Medical Association. This study has been approved by the Medical Ethics Committee of Qilu Hospital of Shandong University. The patients/participants provided their written informed consent to participate in this study. Written informed consent was obtained from the individual(s) for the publication of any potentially identifiable images or data included in this article.

## 2 Case description

### 2.1 Case report 1

**FIGURE 1:**
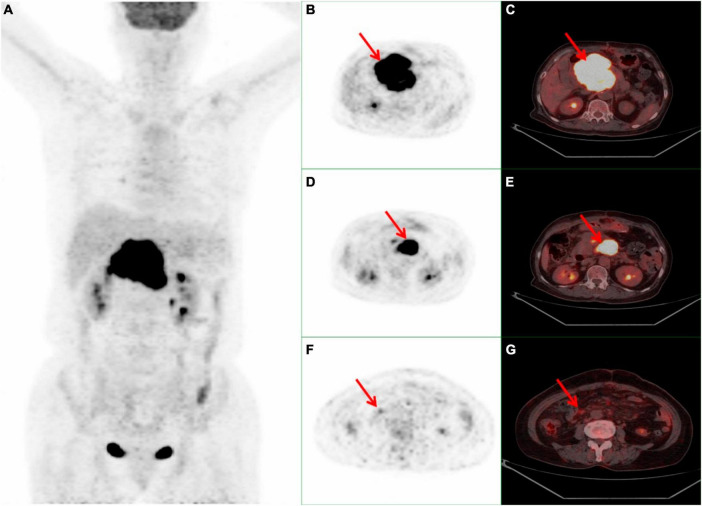
Whole body ^18^F-FDG PET/CT scan of a 70-year-old female with PPL. **(A)** MIP demonstrated a hypermetabolic lesion in the superior abdominal. **(B,C)** A 9.5 × 8.3 × 7.1 cm high FDG-avid irregular occupying at the head of the pancreas with a SUVmax 22.4 (red arrow). **(D, E)** Peripancreatic hypermetabolic enlarged lymph nodes (red arrow). **(F, G)** Suspicious mild hypermetabolic retroperitoneal lymph node with (red arrow). PPL, primary pancreatic lymphoma; ^18^F-FDG PET/CT, ^18^F-fluorodeoxyglucose positron emission tomography/computed tomography; MIP, maximum intensity projection; SUVmax, maximal standardized uptake value.

A 70-year-old female presented with a one-month history of unexplained pain below the xiphoid process. She had undergone a myomectomy three decades earlier. Abdominal contrast-enhanced computed tomography (CT) disclosed a slightly hypodense space-occupying lesion within the pancreatic head, accompanied by mild dilatation of the pancreatic duct but without bile duct dilatation. The enhanced scan exhibited relatively modest enhancement. No irregularities emerged during tumor marker assessments, including CA-199, CEA, and CA-125. Laboratory analyses indicated hepatic function impairment (ALT: 539°U/L, AST: 472°U/L, GLD: 140.5°U/L, γ-GT: 615°U/L, ALP: 821°U/L, TBIL: 178.9°μmol/L, DB: 131.5°μmol/L, IBIL: 47.4°μmol/L). In order to pursue further assessment, the patient underwent an ^18^F-FDG PET/CT examination. The maximum intensity projection (MIP) of the ^18^F-FDG PET/CT divulged a hypermetabolic lesion within the upper abdomen (Figure 1A). A 9.5 × 8.3 × 7.1 cm irregular lesion situated at the pancreatic head exhibited noteworthy FDG uptake, characterized by a maximal standardized uptake value (SUVmax) of 22.4 (Figures 1B–E). Mild to moderately FDG-avid peripancreatic lymph nodes, displaying indistinct boundaries with the pancreatic mass (Figures 1B–E). Additionally, mildly hypermetabolic retroperitoneal lymph nodes were detected (Figures 1F, G). Subsequent to these findings, the patient underwent a pancreatic tumor biopsy and gastrojejunostomy under general anesthesia. The conclusive histopathological diagnosis unveiled diffuse large B-cell lymphoma of the pancreatic head, identified as the non-germinal center type. Subsequently, this patient commenced a low-dose CHOP chemotherapy regimen combined with rituximab (R-miniCHOP). Encouragingly, the patient displayed commendable tolerance and remained devoid of pronounced adverse reactions. At present, the patient is engaged in the sixth cycle of chemotherapy.

### 2.2 Case report 2

**FIGURE 2:**
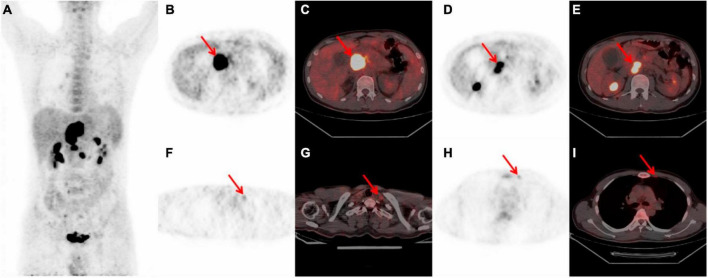
Whole body ^18^F-FDG PET/CT scan of a 31-year-old male with PPL. **(A)** MIP showed a hypermetabolic mass in the superior abdominal and peripheral lesions. And mild hypermetabolic of FDG were observed in the thoracic spine. **(B, C)** A 5.5 × 5.4 cm high FDG-avid mass at the head and neck of the pancreas with SUVmax 22.0 (red arrow). **(D,E)** Hypermetabolic lymph nodes in the posterior of pancreatic region (red arrow). **(F,G)** Left clavicular region lymph node with mild FDG hypermetabolism (red arrow). **(H, I)** Suspicious mild hypermetabolism within the left internal mammary lymph nodes (red arrow). PPL, primary pancreatic lymphoma; ^18^F-FDG PET/CT, ^18^F-fluorodeoxyglucose positron emission tomography/computed tomography; MIP, maximum intensity projection; SUVmax, maximal standardized uptake value; CT, computed tomography.

A 31-year-old male presented with a persistent post-prandial stomach flatulence for a duration of 2°months, accompanied by abdominal pain lasting for 1°month. The abdominal pain exhibited mild tenderness and was not concomitant with any additional discomfort. The patient bore a medical history marked by ankylosing spondylitis and hemorrhoids. The scrutiny of tumor markers rendered abnormal outcomes: sSCC-Ag at 1.800°ng/ml, ferritin at 459.10°ng/ml, CA125 at 171.00°U/ml, and CA19-9 at 12.00°U/ml. Abdominal ultrasonography revealed no aberrations, while electronic gastroscopy indicated chronic non-atrophic gastritis. The abdominal contrast-enhanced CT unveiled occupying lesions within the pancreas, coupled with lymph node enlargement within the retroperitoneal space. Subsequent ^18^F-FDG PET/CT illustrated a hypermetabolic lesion situated in the upper abdomen, with peripheral subsidiary lesions evident in the maximum intensity projection (MIP) (Figure 2A). Notably, the thoracic spine also exhibited mild hypermetabolism (Figure 2A). ^18^F-FDG PET/CT delineated a conspicuously intense hypermetabolic lesion (SUVmax 22.0) situated at the head and neck of the pancreas. Concurrently, hypermetabolic lymph nodes in the posterior pancreatic region were observed (Figures 2B–E). Additionally, mild hypermetabolic (SUVmax 2.3) lymph nodes were found in left clavicular region and left internal mammary (Figures 2F–I). Subsequent CT-guided puncture biopsy of the pancreas yielded a diagnosis of non-Hodgkin T-cell lymphoma. Bone marrow puncture results were normal. Upon diagnosis, the patient initiated chemotherapy, commencing with one cycle of CHOP-E, followed by six cycles of CHOPE combined with chidamide, and culminating in one cycle of cyclophosphamide and etoposide. Following the administration of five chemotherapy cycles, the patient experienced neutropenia and fever, with a neutrophil count of 1.24 × 10^9^/L and a body temperature of 37.8°C. The condition improved in response to anti-infection treatment. At present, the patient is undergoing the eighth cycle of chemotherapy.

## 3 Discussion

We present two cases of primary pancreatic lymphoma diagnosed with ^18^F-FDG PET/CT assistance in this article. For further investigation, we conducted an exhaustive literature review encompassing publications until July 2023, sourced from PubMed, Embase, and Web of Science databases. The predetermined inclusion criteria comprised the following aspects: Publications authored in the English language. Works involving human subjects. Cases of PPL that were accompanied by ^18^F-FDG PET/CT examination data.

The systematic search of the literature, a cumulative total of 19 articles (including 23 instances of PPL) (7–25) met the stipulated inclusion criteria. Incorporating our own two cases, the collective review encapsulated a total of 25 instances of PPL (including 31 lesions). The data assimilation process encompassed a spectrum of aspects, including age, gender, presenting symptoms, pathological findings, treatment modalities, prognosis, laboratory examination results, as well as morphological and PET imaging attributes of the respective cases. We conducted a comprehensive analysis of the data in the literatures and a descriptive statistical analysis of the counting data recorded in the literature. Data sets exhibiting a normal distribution were represented statistically in the format of mean ± standard deviation, whereas those deviating from the norm were expressed in the format of M (P25, P75).

The clinical manifestations of primary pancreatic lymphoma lack specificity, the clinical datas of our PPL cases are summarized in Table 1. Among the cases, a predominant male preponderance was evident, exhibiting a male-to-female ratio of 2.125:1. A total of 24 cases were documented with clinical symptoms, with gastrointestinal symptoms, particularly abdominal pain, prevailing as the most commonly reported clinical manifestation (15 out of 24 cases, 62.5%). All cases in our study were subjected to pathological confirmation, and DLBCL emerged as the principal subtype (40%). Our results are consistent with previous studies (26, 27).

**TABLE 1:** Summary of clinical data.

References	Age/sex	Symptoms	Pathology	Treatment	Status/FU (month)
Yoon et al. (8)	67/M	Bellyache, cholecystalgia	DLBCL	LC and pancreatectomy combined with splenectomy	/
Abe et al. (9)	67/M	epigastrium tenderness, Indigestion, fever.	DLBCL	/	/
Savari et al. (10)	56/M	Asymptomatic	B-cell lymphoma	Surgical treatment and chemotherapy	/
Yadav et al. (11)	53/F	Bellyache	DLBCL	/	D,1 mo
	55/F	Bellyache	DLBCL	Chemotherapy and immunotherapy	A, 14 mo
Nakamura et al. (12)	27/F	Bellyache, jaundice	B-cell lymphoma	/	/
León-Asuero-Moreno et al. (13)	55/M	Asymptomatic	MALT Lymphoma	Radiotherapy	A, 7 mo
Cagle et al. (14)	71/F	Bellyache, postprandial distress	B-cell lymphoma	Chemotherapy (R-CHOP)	/
Jonnalagadda et al. (15)	36/F	Bellyache, lumbago, nausea	Anaplastic large cell lymphoma	Chemotherapy (EPOCH)	A, 1 mo
Yamai et al. (16)	52/M	Bellyache, nausea	Mantle cell lymphoma	Chemotherapy (R-THP-COP)	A, 24 mo
Shapira et al. (17)	73/M	Asymptomatic	Mantle cell lymphoma	Chemotherapy (R-THP-COP)	A, 96 mo
Liu et al. (18)	4/M	Bellyache, jaundice	Burkitt’s lymphoma	Chemotherapy (COP)	/
Okamoto et al. (19)	52/M	Bellyache, jaundice	DLBCL	Surgical treatment and chemotherapy (CHOP)	A, 10 mo
Bozzoli et al. (20)	67/M	Asymptomatic	Follicular lymphoma	Chemotherapy	A, 15 mo
	70/M	Asymptomatic	Follicular lymphoma	Chemotherapy	/
Zafar et al. (21)	15/M	Asymptomatic	DLBCL	Immunochemotherapy (R-CHOP)	A, 60 mo
Boninsegna et al. (22)	57/M	Bellyache, pectoralgia, melena, dizzy	DLBCL	Immunochemotherapy (R-EPOCH)	A, 3 mo
Anand et al. (23)	51/M	/	DLBCL	/	/
Wang et al. (24)	64/M	Bellyache	Lymphoma	/	/
	72/M	Bellyache, jaundice	Lymphoma	Chemotherapy	/
	69/F	Fever	Lymphoma	Chemotherapy	/
Nakaji et al. (25)	69/M	Asymptomatic	DLBCL	Chemotherapy	/
Sadot et al. (26)	57/F	Bellyache, fever	B-cell lymphoblastic lymphoma	Chemotherapy (R-hyper-CVAD) + radiotherapy	A, 6 mo
Present case 1	70/F	Unexplained hidden pain under the xiphoid process	DLBCL	Chemotherapy (R-miniCHOP)	A, 7 mo
Present case 2	31/M	Bellyache, Stomach flatulence	Non-hodgkin T-cell Lymphoma	Chemotherapy (CHOP-E,CHOPE + chidamide and cyclophosphamide + etopogon)	A, 27 mo

M, male; F, female; Y, yes; N, no; FU, follow up; mo, month; A, alive; D, died; DLBCL, diffuse large B-cell lymphoma; LC, laparoscopic cholecystectomy; CT, computer tomography; MRI, magnetic resonance imaging; CHOP, cyclophosphamide; doxorubicin; vincristine; and prednisone; R-CHOP, rituximab combined CHOP; EPOCH, etoposide; prednisone; vincristine; cyclophosphamide; and doxorubicin; R-THP-COP, rituximab; pirarubicin; cyclophosphamide; vincristine; and prednisolone; COP, cyclophosphamide; vincristine; and prednisolone; EPOCH, rituximab combined EPOCH; R-hyper-CVAD, rituximab plus fractionated cyclophosphamide; vincristine; doxorubicin and dexamethasone alternating with high-dose methotrexate-cytarabine; R-MiniCHOP, low-doesCHOP chemotherapy regimen-rituximab combination; CHOP-E, CHOP plus etoposide.

Past investigations revealed laboratory evaluations often disclose abnormal liver function and escalated pancreatic enzyme levels, whereas elevated tumor markers assume less prevalence. Remarkably, certain patients showcase an increase in CA199 (4, 26, 28, 29). Aberrant liver function might be attributed to a sizable mass situated at the pancreatic head, causing obstructions within the common bile duct. In Our cases, sixteen cases possessed recorded laboratory test results, as detailed in Table 2, 80% (4 out of 5) of our cases characterized by irregular liver function had lesions located within the pancreatic head. And three cases evidenced elevated tumor markers (including CEA, CA199, and CA125), two cases demonstrated heightened levels of pancreatic enzymes (lipase and amylase).

**TABLE 2:** Summary of laboratory test.

References	Age/sex	Tumor marker	Liver function and pancreatin
Yoon et al. (8)	67/M	/	/
Abe et al. (9)	67/M	/	/
Savari et al. (10)	56/M	Tumor markers such as CEA and CA19-9 are normal.	/
Yadav et al. (11)	53/F	CA19-9 was within the normal range (<0.8°U/mL).	Lipase was elevated to 666 IU/L.
	55/F	/	Serum lipase level was within the normal range.
Nakamura et al. (12)	27/F	/	/
León-Asuero-Moreno et al. (13)	55/M	CEA showed a high value at 13.0°ng/dl (&3.5), and the NSE was elevated slightly.	/
Cagle et al. (14)	71/F	/	/
Jonnalagadda et al. (15)	36/F	/	/
Yamai et al. (16)	52/M	Tumor markers were normal (AFP, Ca 125, CA 19-9, and CEA).	Liver function tests were normal.
Shapira et al. (17)	73/M	Tumor markers (CEA, CA19-9 and DUPAN-2) were within the normal range.	Normal
Liu et al. (18)	4/M	/	TB: 4.4 mg/dl (normal, 0/2–1 mg/dl); DB: 3.9 mg/dl (normal, 0–0.2 mg/dl); liver function tests were abnormal (including ALT, AST, ALP). Blood amylase and lipase levels were also elevated; LDH was mildly elevated at 280°U/l (normal 60–225°U/l).
Okamoto et al. (19)	52/M	CA199 increased (215.8).	TB increased (73.9).
Bozzoli et al. (20)	67/M	CEA and CA19-9 were within the normal range.	/
	70/M	CEA, and CA19-9 were unremarkable	/
Zafar et al. (21)	15/M	Tumor markers included CA19-9, AFP and CEA were within normal ranges.	Abnormal liver function tests (AST 73°UI/l, ALT 144°UI/l, ALP: 583°UI/l, TB: 10.61 mg/dl), elevated LDH (504°UI/l) and β2-MG (3.2 mg/l).
Boninsegna et al. (22)	57/M	CEA, CA19-9, and AFP were all negative.	Liver function tests and lipase level were within normal limits.
Anand et al. (23)	51/M	/	/
Wang et al. (24)	64/M	/	/
	72/M	/	/
	69/F	/	/
Nakaji et al. (25)	69/M	Tumor-marker panel examination was normal.	/
Sadot et al. (26)	57/F	/	LDH 421 U/L (normal range 119–229 U/L).
Present case 1	70/F	Tumor-marker examination was normal (CA199:7.56°u/ml, CA125:17.2°u/ml).	Liver function was abnormal (ALT: 539U/L, AST: 472U/L, GLD: 140.5°U/L, γ-GT: 615°U/L, ALP: 821°U/L, TB: 178.9°umol/L, DB: 131.5°umol/L, IBIL: 47.4°umol/L, prealbumin: 11.4°mg/dl, TBA: 117.1°umol/L).
Present case 2	31/M	SCC-Ag was 1.800°ng/ml, ferritin was 459.10°ng/ml, CA125 was171.00°U/ml, CA199 was 12.00°u/ml.	/

CA19-9, carbohydrate antigen 19-9; CA125, carbohydrate antigen 125; CEA, carcinoembryonic antigen; NSE, neuron-specific enolase; AFP, alpha-fetoprotein; SCC-Ag, Squamous cell carcinoma associated antigen; TB, total bilirubin; DB, direct bilirubin; ALT, Alanine aminotransferase; AST, Aspartate aminotransferase; ALP, alkaline phosphatase; LDH, lactate dehydrogenase; β2-MG, beta2- Microglobulin; GLD, glutamate dehydrogenase; γ-GT, γ-glutamyl transpeptidase; TBA, total bile acid.

In the realm of treatment, chemotherapy represents the primary modality for PPL (30). Solely relying on surgical intervention typically yields inferior prognoses compared to cases treated exclusively with chemotherapy. Consequently, surgery is better positioned as a diagnostic or palliative strategy (26, 31, 32). The post-chemotherapy prognosis for PPL is generally promising (26, 33), while advanced age (≥60 years), female gender, unmarried status, higher staging, and absence of chemotherapy emerge as adverse prognostic indicators for PPL (31, 32). Relapses within PPL predominantly manifest at distant sites, with pancreatic relapses being comparatively infrequent. Remarkably, a risk of central nervous system (CNS) relapse is also underscored (26, 32). In our cases, a Eighteen cases underwent chemotherapy, with the rituximab-combined CHOP regimen (R-CHOP) standing out as the prevailing therapeutic approach. Only one patient succumbed to septic shock 5°weeks post-diagnosis, while the remaining cases continue to exhibit vitality.

^18^F-fluorodeoxyglucose positron emission tomography/computed tomography (^18^F-FDG PET/CT) offers the dual advantage of functional and morphological imaging. On CT scans, discerning a relatively sizeable mass in the pancreatic head devoid of pancreatic duct dilation reinforces the suspicion of PPL. Notable features include a pancreatic duct that generally remains either normal or exhibits mild displacement and constriction and invasive pancreatic masses infiltrating the pancreatic borders, along with enlarged lymph nodes below the renal vein level. Furthermore, untreated PPL seldom demonstrates calcification or necrosis. Bile duct dilation attributed to obstruction predominantly characterizes PPL situated at the pancreatic head (34, 35). The prevailing CT characteristics gleaned from the PPL cases incorporated within this study correspond with localized, substantial, low-density masses, with only three instances featuring mild pancreatic duct dilation. A substantial proportion (19 out of 25 cases, 76%) exhibited solitary lesions, while the remaining six cases demonstrated the presence of dual lesions. Notably, no preferential localization for lesions emerged, with 17 lesions situated in the pancreatic head and neck region, and 14 lesions located within the body and tail of the pancreas. The lesion dimensions were relatively expansive, with the median of maximum diameter recorded in 21 cases is 4.30 (3.15, 7.80) cm. Moreover, two cases of pancreatic head lesions engendered intrahepatic bile duct dilation, aligning seamlessly with prior investigations. The morphological imaging findings for the array of PPL cases are meticulously compiled within Table 3.

**TABLE 3:** Summary of morphological imaging findings.

References	Age/sex	Direct sign	Dilation of pancreatic duct or bile duct
Yoon et al. (8)	67/M	CT, MRI of the abdomen showed cholecystitis with gallstone and incidentally 1.5 cm sized hypodense round mass located at the tail of pancreas.	N
Abe et al. (9)	67/M	CT plain scan: a 5.4-cm lobulated low-density mass in the pancreas.	N
Savari et al. (10)	56/M	Plain CT scan: a 5-cm tumor located in the head of pancreas. Enhanced CT scan: a slight increase in the tumor without encasement of arteries or veins. MRI scan: a mass with homogeneously high signal intensity on T2-weighted images and low signal intensity on T1-weighted images with gadolinium enhancement.	N
Yadav et al. (11)	55/F	Plain CT scan: a tumor lobulated in the posterior aspect of the pancreatic tail, which encases multiple mesenteric vessels and displaces the left adrenal gland.	N
	55/F	/	/
Nakamura et al. (12)	27/F	Plain CT scan: a mass homogenously isodense to pancreas with HU value 33. Enhanced CT scan: on pancreatic and portal venous phase mass were homogenously hypoenhancing as compared to rest of pancreas.	Mass causing obliteration of proximal and mid CBD with upstream dilated common hepatic duct and bilobar moderate intrahepatic biliary radicle dilation.
León-Asuero-Moreno et al. (13)	55/M	Plain CT scan and enhanced CT scan: masses showed iso-attenuated patterns even after an enhanced phase, and so it was difficult to recognize them clearly.	N
Cagle et al. (14)	71/F	/	/
Jonnalagadda et al. (15)	36/F	Enhanced CT: a large hypodense lesion centered within the junction of the neck/body of the pancreas with mild dilation of the pancreatic duct.	Mild dilation of the pancreatic duct.
Yamai et al. (16)	52/M	Plain and enhanced CT scan: A low attenuation mass with no dilation of the pancreatic duct.	N
Shapira et al. (17)	73/M	Enhanced CT scan: masses with faint enhancement, while atrophy of the pancreatic parenchyma or dilation of the main pancreatic duct was not observed.	N
Liu et al. (18)	4/M	Enhanced CT scan: two low attenuation masses in the pancreas and mild intrahepatic bile duct dilation.	Mild intrahepatic bile duct dilation.
Okamoto et al. (19)	52/M	/	/
Bozzoli et al. (20)	67/M	Plain and enhanced CT: a slowly-enhancing mass in the pancreatic head. Enhanced MRI: a heterogeneous pancreatic mass and mesenteric lymphadenopathy with abnormal diffusion restriction.	N
	70/M	Plain and enhanced CT: a slowly enhancing mass in the pancreatic body and mildly enlarged. mesenteric lymph nodes.	N
Zafar et al. (21)	15/M	Plain and enhanced CT: low density tumor, heterogeneously enhancing and obstructing the common bile duct and the main pancreatic duct, many centimetric peripancreatic lymphadenopathies and normal spleen and liver.	N
Boninsegna et al. (22)	57/M	CT scan and MRCP: a large, heterogeneous, peripherally enhancing mass, with central necrosis, measuring 8.3 × 5.3 cm, centered on the tail of the pancreas. The mass was extending into the splenic hilum and invading the left kidney. Retroperitoneal lymphadenopathy and multiple adjacent soft tissue nodules were noted along with the involvement of the left adrenal gland.	N
Anand et al. (23)	51/M	Plain and enhanced CT: a large isodense mass in the body-head of the pancreas, the arteries and veins are encased but not infiltrated. The main pancreatic duct is not enlarged.	N
Wang et al. (24)	64/M	Plain and enhanced CT: a small, well-defined relatively homogenous, hypovascular mass.	N
	72/M	Plain and enhanced CT: a large heterogeneous mass in the pancreatic head. Despite the large size and localization in the pancreatic head, no pancreatic duct dilatation is seen. Vascular encasement is seen without any evidence of occlusion.	N
	69/F	/	/
Nakaji et al. (25)	69/M	Plain CT: low-density mass.	N
Sadot et al. (26)	57/F	Enhanced CT scan: a solid mass in the pancreatic body without vascular occlusion. The superficial and mediastinal lymph nodes were not enlarged. MRI: a mass that exhibited homogenous low signal intensity on T1- and T2-weighted images (T1WI and T2WI, respectively) and high signal intensity on DWI.	A section of the duct in the tail of pancreas was dilated
Present case 1	70/F	Enhanced CT scan: a slightly low-density mass at the head of the pancreas, with mild dilation of the pancreatic duct.	Mild dilation of the pancreatic duct
Present case 2	31/M	Plain and enhanced CT scan: a low density masses at the head and neck of the pancreas.	N

M, male; F, female; N, no; CT, computer tomography; MRI, magnetic resonance imaging; MRCP, magnetic resonance cholangiopancreatography; HU, Hounsfield unit; CBD, common bile duct.

The most distinctive PET hallmark of PPL resides in the conspicuous hypermetabolic lesion discernible within the pancreas. Among the accumulated cases, a total of 16 lesions featured the documentation of the maximal standardized uptake value (SUVmax), yielding an average SUVmax of 14.37 ± 7.95, with a range spanning from 4.10 to 28.00. Furthermore, eleven instances evidenced the involvement of contiguous lymph nodes, of which five cases exhibited evidence of distant dissemination, encompassing lymph nodes in various locations including the splenic hilum, left kidney hilum, lower esophageal region, left supraclavicular region, and left internal mammary region. Notably, one case disclosed FDG uptake within the left adrenal gland, attributed to tumor invasion. The PET imaging findings for the array of PPL cases are meticulously compiled within Table 4.

**TABLE 4:** Summary of ^18^F-FDG-PET/CT findings.

References	Number of lesions	Size (cm)	Site	SUVmax	Involvement of lymph nodes and organs	^18^F-FDG PET/CT after treatment
Yoon et al. (8)	1	1.5	Tail of Pancreas	/	N	/
Abe et al. (9)	1	4.5	Head of pancreas	/	N	/
Savari et al. (10)	2	5	Head and body of pancreas	8.67(Head of pancreas)	N	/
Yadav et al. (11)	2	3.3, 4.2	Head and neck of pancreas	/	Y (mesenteric lymph nodes)	/
	1	11	Tail of pancreas		N	/
Nakamura et al. (12)	1	6.6	Head and neck of pancreas	13	N	/
León-Asuero-Moreno et al. (13)	2	3, 1	Head and body of pancreas	6.01, 5.35	N	Y
Cagle et al. (14)	1	/	Body of pancreas	11.66	Y (posterior pancreatic region, splenic hilum and left kidney hilium lymph nodes)	Y
Jonnalagadda et al. (15)	1	/	Body of pancreas	/	Y (mesenteric lymph nodes)	Y
Yamai et al. (16)	1	15	Tail and body of pancreas	11.93	N	Y (recurrence)
Shapira et al. (17)	1	/	Head of pancreas	5.4	N	Y (recurrence)
Liu et al. (18)	2	4.3,2.8	Head and body of pancreas	/	N	Y
Okamoto et al. (19)	1	3.9	Head of pancreas	22.8	N	/
Bozzoli et al. (20)	1	4	Head of pancreas	6.7	Y (paraaortic lymph nodes)	/
	1	2.8	Body of pancreas	4.1	Y (abdominal, lower esophageal, and left supraclavicular lymph nodes).	/
Zafar et al. (21)	2	4	Head and tail of Pancreas	/	Y (hepato-gastric ligament lymph nodes)	Y
Boninsegna et al. (22)	1	8.3	Tail of pancreas		Y (lymph nodes in the abdomen).	Y
Anand et al. (23)	1	/	Head of pancreas	/	N	/
Wang et al. (24)	1	/	Head of pancreas	20	N	/
	1	/	Head of pancreas	28	Y (left adrenal).	/
	2	/	Body and tail of pancreas	/	N	Y
Nakaji et al. (25)	1	7.3	Head of pancreas	22.8	N	Y
Sadot et al. (26)	1	10	Body of pancreas	19.11	Y (peripancreatic lymph nodes)	/
Present case 1	1	9.5	Head of pancreas	22.4	Y (peripancreatic and retroperitoneal lymph nodes)	N
Present case 2	1	5.5	Head and neck of pancreas	22	Y (Peripancreatic, retroperitoneal, left supraclavicular and left internal mammary lymph nodes)	N

Y, yes; N, no; SUVmax, maximal standardized uptake value max.

The versatility of ^18^F-FDG PET/CT extends to its ability to discern glycometabolic changes within lesions prior to observable anatomical alterations, rendering it an invaluable asset for baseline assessment and the assessment of lymphoma treatment efficacy (5, 6, 36). In our study, two instances (9, 20) unveiled smaller pancreatic lesions that evaded detection via CT and MRI, accompanied by comparatively lower FDG uptake than their larger counterparts. Notably, among our cases, ten subjects underwent post-treatment ^18^F-FDG PET/CT evaluations, uncovering two cases of recurrence within the pancreas and peripancreatic lymph nodes. Following a change in chemotherapy regimen, subsequent scans signaled remission. The remaining cases indicated a state of remission on ^18^F-FDG PET/CT scans post-treatment.

^18^F-fluorodeoxyglucose positron emission tomography/computed tomography (^18^F-FDG PET/CT)’s significance in staging diverse lymphoma categories, particularly DLBCL, is profound. Moreover, for indolent lymphomas, ^18^F-FDG PET/CT emerges as a pivotal tool for identifying histological transformations from low-grade indolent lymphomas to more aggressive high-grade forms (36). One of the rationale behind ^18^F-FDG PET/CT’s superior staging potential compared to contrast-enhanced CT lies in its heightened sensitivity in detecting bone marrow involvement. Nevertheless, consensus remains elusive regarding whether diffuse FDG uptake in bone marrow should be construed as a definitive marker of bone marrow involvement (37). Prior investigations have suggested that diffuse bone marrow uptake in DLBCL signifies an elevated likelihood of bone marrow invasion. Conversely, diffuse bone marrow uptake in Hodgkin lymphoma (HL) is more likely indicative of bone marrow inflammatory changes (38). Nonetheless, as these studies feature relatively modest sample sizes, further research is warranted to elucidate this matter. An illustrative case within our study exhibited mild diffuse hypermetabolism in the thoracic vertebrae, despite normal findings from a bone marrow biopsy.

According to the WHO’s classification, PPL can indeed entail contiguous region lymph node involvement and distant spread, while the core of the lesion resides within the pancreas. Our study underscores that PPL commonly involves contiguous region lymph nodes, but distant spread is less prevalent. The distinction between PPL and secondary pancreatic lymphoma hinges on the status of lesions beyond the pancreas, with ^18^F-FDG PET/CT emerging as pivotal in this regard. ^18^F-FDG PET/CT effectively detects the primary lesion and distant spread of PPL, proficiently demarcating it from secondary pancreatic lymphoma. Compared with the ^18^F-FDG PET/CT findings of PPL, there are more lesions in other parts of secondary pancreatic lymphoma and higher uptake of FDG. Intriguingly, the pancreatic lesions in secondary pancreatic lymphoma are similar to those of PPL, characterized predominantly by sizeable hypermetabolic masses (39).

The precise differentiation between PPL and pancreatic carcinoma assumes paramount importance. Given the relatively favorable prognosis associated with PPL following chemotherapy (26, 33), accurate distinction from pancreatic carcinoma holds potential to avert unnecessary surgical interventions. At times, PPL proves elusive to traditional diagnostic techniques like computed tomography (CT) or magnetic resonance imaging (MRI). Of the cases assimilated within this study, 14 instances garnered a preliminary diagnostic conclusion via traditional imaging (CT or MRI) prior to pathological confirmation. Regrettably, a mere three cases received a lymphoma diagnosis, yielding a diagnostic accuracy of 21.4%, and predominantly manifesting as misdiagnoses spanning pancreatic cancer or malignant pancreatic masses. Pancreatic carcinoma exhibits a higher prevalence among males, with its zenith incidence occurring beyond the age of 70 years (40). In accordance with extant literature reports (27), PPL also displays a male predilection, yet its onset is characteristically earlier than that of pancreatic carcinoma, with a median age of 67. Additionally, a study (41) uncovered elevated CA199 levels in instances of pancreatic carcinoma (406.81 ± 352.09), while liver function (ALT, AST, ALP, etc.) and pancreatic enzymes were generally in a normal range. This stands in contrast to the laboratory findings in cases of PPL. Through an exhaustive scrutiny of antecedent literature (42, 43) including 230 instances of pathologically confirmed pancreatic carcinoma, it is established that pancreatic carcinoma predominantly manifests as hypoattenuated lesions during CT enhancement scans, occasionally displaying isodensity. The typical maximum diameter of isodensity pancreatic carcinoma lesions seldom exceeds 2 cm, while their hypoattenuated counterparts demonstrate an average maximum diameter of approximately 3 cm. Moreover, characteristic imaging attributes of pancreatic carcinoma encompass pancreatic ductal truncation, dilation of pancreatic ducts, and cholangiectasis (42, 44). Contrasting with the PPL cases expounded within this exposition, it becomes evident that PPL lesions generally exhibit relatively greater dimensions. Notably, the dilation of the bile duct and pancreatic duct is a rarity in PPL cases; however, akin to pancreatic carcinoma, both maladies predominantly showcase hypoattenuated lesions upon contrast-enhanced CT imaging.

As per antecedent investigations, both pancreatic carcinoma and PPL predominantly exhibit hypermetabolic lesions on ^18^F-FDG PET/CT scans. In the study by Moon (45), the SUVmax of 21 pancreatic carcinoma lesions registered at 6.8 ± 3.0, with a range spanning 2−12. An examination by Zhang et al. (41) on 40 patients with pancreatic carcinoma unveiled an average SUVmax of 7.30 ± 3.21 for 40 pancreatic carcinoma lesions. In a study conducted by Lee et al. (46) encompassing 87 cases of pancreatic carcinoma, the median SUVmax for pancreatic carcinoma lesions was 4.4, oscillating between 2.1 and 17.3. Drawing from the aforementioned investigations, when juxtaposed against the SUVmax value of PPL documented within our discourse (14.37 ± 7.95), it becomes apparent that the SUVmax of pancreatic carcinoma cases tends to be lower in comparison to those of PPL cases. Furthermore, the metastatic dissemination patterns for these two conditions deviate. Predominantly, pancreatic adenocarcinoma metastasizes to the liver, subsequently to the peritoneum, abdominal lymph nodes, and harbors a notable likelihood for lung metastasis. As a rule, pancreatic adenocarcinoma engages in multi-organ metastasis (47). In our analysis, PPL demonstrates a scarcity of distant dissemination, with sporadic instances of distant lymph node metastasis, thereby markedly diverging from the distant metastatic mode typified by pancreatic adenocarcinoma.

In summation, ^18^F-FDG PET/CT, functioning as a non-invasive functional imaging modality, assumes a pivotal role in diagnosing, initially assessing, and gauging the efficacy of treatment for PPL. Concurrently, the amalgamation of ^18^F-FDG PET/CT image attributes with clinical profiles and laboratory analyses possesses the potential to furnish more insightful information, facilitating the discrimination between PPL and pancreatic carcinoma.

## 4 Scope statement

Primary pancreatic lymphoma (PPL) constitutes a rare malignancy, with an incidence rate of less than 2% among extranodal non-Hodgkin lymphomas in populations with good immunocompetent. Owing to its rarity and a lack of distinct clinical manifestations, PPL poses a considerable diagnostic challenge prior to histopathological assessment, frequently leading to misdiagnoses akin to other pancreatic space-occupying lesions, such as pancreatic carcinoma.

^18^F-fluorodeoxyglucose positron emission tomography/computed tomography (^18^F-FDG PET/CT) has emerged as a widely embraced functional imaging technique. It assumes a pivotal role in discerning differential diagnoses, facilitating staging procedures, and monitoring the trajectory of lymphomas. However, owing to the infrequent occurrence of PPL, there exists a dearth of literature detailing the distinctive ^18^F-FDG PET/CT features pertinent to PPL.

The purpose of this article is to explore the potential value of ^18^F-FDG PET/CT in the diagnosis and treatment framework of PPL. By reporting two cases and literature review, a total of 25 cases (including 31 lesions) assessed by ^18^F-FDG PET/CT before treatment were included. The results of this study reveal that ^18^F-FDG PET/CT has important value in the diagnosis of PPL and the differential diagnosis between PPL and pancreatic carcinoma, and can effectively evaluate the therapeutic effect of PPL.

## Data availability statement

The original contributions presented in this study are included in this article/supplementary material, further inquiries can be directed to the corresponding author.

## Ethics statement

The studies involving humans were approved by the Medical Ethics Committee of Qilu Hospital of Shandong University. The studies were conducted in accordance with the local legislation and institutional requirements. The participants provided their written informed consent to participate in this study. Written informed consent was obtained from the individual(s) for the publication of any potentially identifiable images or data included in this article.

## Author contributions

JW: Conceptualization, Writing – original draft, Writing – review & editing. YZ: Conceptualization, Data curation, Writing – original draft, Writing – review & editing. HL: Formal Analysis, Investigation, Methodology, Writing – review & editing. JZ: Formal Analysis, Investigation, Methodology, Writing – review & editing. XL: Project administration, Supervision, Writing – original draft, Writing – review & editing.
